# Incorporating a Growth Mindset Model Into Medical Education and Enhancing the Complex Problem-Solving Abilities and Mental Resilience of Medical Students and Residents

**DOI:** 10.7759/cureus.67294

**Published:** 2024-08-20

**Authors:** Ning Zhang

**Affiliations:** 1 Department of Geriatrics, Peking Union Medical College Hospital, Peking Union Medical College, Chinese Academy of Medical Sciences, Beijing, CHN

**Keywords:** pritzker school of medicine, medical education research, the university of chicago, international medical educators program, growth mindset

## Abstract

Peking Union Medical College Hospital and the University of Chicago have established a robust partnership characterized by collaboration and camaraderie. These two institutions engage in productive interactions and exchanges concerning resident physician training, teaching methodologies, and specialized academic collaborations. In July 2019, Peking Union Medical College Hospital sent senior physicians to the University of Chicago for their inaugural participation in the International Medical Educators Program (IMEP). In 2020, due to the COVID-19 pandemic, the IMEP transitioned to an online training format. The IMEP course for 2024 features a hybrid model of both offline and online instruction, marking the return to in-person teaching for the first time since the onset of the pandemic. This year's curriculum primarily emphasizes 'Curriculum Development for Medical Education: A Six-Step Approach.' Furthermore, the organizers have planned a diverse array of teaching topics, including Learning Theory, Clinical Competence Assessment, Outpatient Teaching Skills, Teaching on the Fly, Residents as Teachers, Bedside Teaching Skills, Coaching in Medical Education, Online Teaching Skills, Peer Coaching, and Growth Mindset in a Fixed Mindset Culture. Among the courses that significantly broadened my perspective and deepened my understanding, the lecture delivered by Professor James N. Woodruff from the Pritzker School of Medicine at the University of Chicago, titled 'Growth Mindset in a Fixed Mindset Culture,' left the most profound impression on me.

The development of a growth mindset is essential for medical students. Professor Woodruff underscored this point in the course "Growth Mindset in a Fixed Mindset Culture," emphasizing that the future of medical practice will be characterized by complex and dynamic challenges. In this context, errors and mistakes are unavoidable, highlighting the need to shift focus from static abilities to continuous growth. To date, the growth mindset framework established by the Pritzker School of Medicine Well-Being Committee has yielded remarkable results.

The successful experience of the University of Chicago Pritzker School of Medicine has prompted me to conduct a more in-depth analysis of our own practices. Specifically, within the Department of Internal Medicine at Peking Union Medical College Hospital, we have encountered some challenges in the standardized training of residents in recent years. Additionally, there has been a notable increase in the emotional challenges faced by residents. Consequently, it is essential to draw lessons from the successful practices of the University of Chicago Pritzker School of Medicine and to incorporate growth mindset-related training into our resident training system. This integration aims to enhance residents' abilities to navigate complex situations and solve intricate problems. More importantly, it fosters mental resilience, equipping residents to better manage stress and setbacks. The cultivation of a growth mindset is crucial not only for residents but also for senior physicians, as we all confront the complexities of the medical environment, the uncertainties associated with diseases, and the challenges inherent in our professional development. Effectively addressing these challenges requires the support of a growth mindset.

## Editorial

Peking Union Medical College Hospital and the University of Chicago have developed a robust partnership characterized by cooperation and friendship, both of which were supported and initiated by the Rockefeller Foundation. The two institutions engage in fruitful interactions and exchanges regarding resident physician training, teaching methodologies, and specialist academic collaborations. In July 2019, Peking Union Medical College Hospital sent three senior doctors to the University of Chicago for the inaugural participation in the two-week International Medical Educators Program (IMEP). Beginning in 2020, due to the COVID-19 pandemic, the IMEP transitioned to an online training format lasting three months. By 2023, a total of 25 senior doctors from Peking Union Medical College Hospital had participated in the IMEP training. The IMEP course for 2024 adopts a hybrid model that combines both offline and online instruction, marking the first instance of offline teaching since the COVID-19 pandemic. I am honored to have successfully passed the interview selection process at Peking Union Medical College Hospital and to have been chosen for the 2024 IMEP training program. I attended the University of Chicago to participate in this year’s in-person courses from July 8 to 19, 2024. This year's curriculum primarily focused on 'Curriculum Development for Medical Education: A Six-Step Approach.' Additionally, the organizers have scheduled a diverse array of teaching topics, including Learning Theory, Clinical Competence Assessment, Outpatient Teaching Skills, Teaching on the Fly, Residents as Teachers, Bedside Teaching Skills, Coaching in Medical Education, Online Teaching Skills, Peer Coaching, and Growth Mindset in a Fixed Mindset Culture.

These enriching courses have significantly broadened my perspectives. As a geriatrician in a comprehensive teaching hospital, I have been responsible for various educational roles, including bedside teaching for residents, clinical diagnosis instruction for medical undergraduates, training for physicians seeking further education in the Department of Geriatric Medicine at Peking Union Medical College Hospital, and continuing education for specialists in geriatric medicine. My teaching skills have been shaped by the guidance of senior colleagues as well as my own clinical and instructional experiences. However, I had not systematically studied the theory and practice of adult education or the development of medical education curricula. Through my studies at the University of Chicago, I aimed to evolve from a clinician into a clinician who also serves as a medical educator.

Among the courses that significantly expanded my horizons and enhanced my understanding, the lecture delivered by Professor James N. Woodruff from the Pritzker School of Medicine at the University of Chicago, titled 'Growth Mindset in a Fixed Mindset Culture,' left the most profound impression on me. 

Thinking models, also referred to as implicit ideas or mindsets, encompass the fundamental assumptions individuals hold regarding the malleability of their basic characteristics, such as intelligence and moral character [1]. These thinking patterns primarily consist of two types: fixed mindset and growth mindset. Individuals with a fixed mindset often believe that their intelligence and abilities are innate and immutable. In contrast, those with a growth mindset embrace the belief that abilities can develop, adapt, and be enhanced over time [2]. These fundamentally different modes of thinking lead to varying behavioral responses and are accompanied by distinct emotional experiences when faced with success or failure, thereby exerting multiple influences on emotions, behavior, and physiology [3]. A growth mindset is a crucial cognitive quality that significantly impacts the development of students' social and emotional competencies, playing a vital role in enhancing these abilities. As Dweck, the proponent of the growth mindset theory, stated: "The influence of thinking patterns on people is far greater than people imagine. It is no exaggeration to say that thinking patterns are quietly controlling your life" [2].

Dweck's self-theory posits that an individual's cognitive style constrains their behavior, with different cognitive styles leading to distinct behavioral outcomes [4]. This theory is relevant to various dimensions of social and emotional competencies. Existing research indicates that cognitive patterns can significantly influence creative performance; specifically, individuals with fixed mindsets tend to underperform in creative tasks [5], while creative self-efficacy is positively associated with growth mindsets [6]. Curiosity, a facet of openness, serves as a catalyst for learning behaviors and underpins the generation of concepts and ideas. According to the psychological model of growth mindsets [7], individuals who possess a growth mindset exhibit heightened curiosity towards exploring new experiences. Both curiosity and creativity are manifestations of openness, suggesting a notable connection between growth mindsets and open abilities.

Different thinking patterns form the foundation for individuals to establish goals and manage failure [8]. These varying cognitive approaches influence how individuals respond to challenges, thereby assigning distinct values and meanings to setbacks [9]. This, in turn, can impact an individual's capacity to set achievement goals, complete tasks, and perform emotionally across various domains. Regarding task performance, individuals with a growth mindset tend to adopt a mastery-oriented coping style; they view social challenges as valuable opportunities for self-improvement and skill acquisition, and they find that difficulties motivate them to persist in their efforts. Conversely, individuals with a fixed mindset are more likely to employ a goal-oriented coping style. When faced with setbacks, they often experience feelings of powerlessness, which can lead to learned helplessness. Consequently, they may opt for less challenging tasks to validate their abilities when establishing achievement goals [10]. It is evident from their responses to frustration, goal selection, and task completion that individuals with a growth mindset exhibit greater perseverance, self-control, and a heightened sense of responsibility.

Individuals with a growth mindset recognize the plasticity of the brain, embrace challenges, and are not deterred by failure. They tend to adopt an optimistic explanatory style and experience less psychological pressure in the face of setbacks. This resistance to stress is crucial for psychological resilience, serving as a protective factor that enables individuals to maintain their mental health despite adverse circumstances [11]. It is reasonable to conjecture that a growth mindset fosters and sustains positive emotions by enhancing stress resistance. Research indicates a significant positive correlation between a growth mindset in adolescents and the trait of grit [12], which is associated with emotional regulation. Individuals characterized by high levels of grit demonstrate better self-adjustment, exhibit relatively stable emotions, and maintain more positive feelings and expectations [13]. This relationship may elucidate why a growth mindset is closely linked to subjective well-being [14]. Furthermore, studies have shown that a growth mindset correlates positively not only with positive emotions such as happiness and achievement goals but also with factors like school participation and interpersonal relationships [15]. Those with a growth mindset typically adopt a development-oriented perspective on interpersonal relationships, leading them to employ improvement-focused strategies when faced with relational challenges [16]. Additionally, individuals with a growth mindset are generally less prone to engage in problem behaviors and display reduced hostility toward others [17]. Collectively, this evidence suggests that a growth mindset facilitates enhanced performance in social interactions and collaborations.

Developing a growth mindset is critical for medical students. Professor Woodruff emphasized this in the course "Growth Mindset in a Fixed Mindset Culture," highlighting that complex and dynamic problems will define the future of medical practice. In this context, errors and mistakes are inevitable, necessitating a shift in focus from static abilities to continuous growth. Since 2016, the student affairs team at Pritzker School of Medicine has implemented a growth mindset framework in its personal, professional, and academic development programming. To promote a growth mindset, the student well-being program specifically targets several maladaptive aspects of a compliance culture, including error avoidance and perfectionism. A significant event in the medical school's annual calendar is "I Screwed Up at Pritzker!", where students listen to deans, program directors, and clerkship directors recount their mistakes, emotional responses, and the lessons learned from these experiences. Additionally, another mandatory event for all first-year medical students features seniors, residents, and faculty sharing their personal mental health challenges, discussing the impact of these issues on their personal and professional lives, and exploring potential intervention strategies [18].

To date, the growth mindset framework implemented by the Pritzker School of Medicine Well-Being Committee has produced outstanding results. Over the past decade, Pritzker's unmatched rate in the National Resident Matching Program has been one-third of the national average (2% compared to 6%). Remarkably, Pritzker students accomplish this outcome while applying on average to 30% fewer residency programs than the national average. Additionally, responses from the Association of American Medical Colleges Graduation Questionnaire indicate that students have reported being 'very satisfied' at a rate nearly 20% higher than the national average over the last 10 years [18].

The successful experience of the University of Chicago Pritzker School of Medicine has prompted me to engage in a deeper analysis of our own practices. Specifically, within the Department of Internal Medicine at Peking Union Medical College Hospital, we have encountered some challenges in the standardized training of residents in recent years. Notably, there has been a decline in the clinical fundamentals among young residents, which is accompanied by a decrease in enthusiasm and motivation for clinical rotations. This trend has become particularly pronounced following the conclusion of the COVID-19 pandemic. Additionally, there has been a marked increase in emotional challenges faced by residents. Each year, several residents and graduate students are required to temporarily pause their standardized training due to emotional issues, including anxiety and depression.

Senior doctors born in the 1950s and 1960s grew up during China's challenging economic times, many of whom experienced hunger in their childhood. Having faced such difficulties, they became the first cohort of medical students following China's reform and opening up, as the country resumed college entrance examinations and higher education enrollment. Although their living conditions were inadequate during their residency years, these conditions improved significantly in comparison to the hunger and poverty of their early lives. Consequently, they developed resilience and the ability to endure stress from a young age, fostering what is now referred to as a growth mindset. In contrast, most of today's young residents were born after 2000, growing up during a period of rapid economic expansion following China's accession to the World Trade Organization. They benefited from abundant resources and a nurturing environment, often receiving considerable attention from their parents. However, being predominantly only child, many of them also experience feelings of loneliness and fragility. Upon entering the hospital and commencing their residency, individuals experience significant pressure. They are faced with demanding clinical workloads, challenges in doctor-patient communication, and the hospital's stringent expectations regarding residents' scientific research output. Throughout their prior education in basic and medical training, no courses adequately prepared them to navigate these challenges. The pressures, setbacks, and failures encountered in a complex system comprising hospitals, patients, diseases, colleagues, doctor-patient relationships, and medical frameworks can be overwhelming.

The assessment orientation of hospitals further exacerbates this issue. In China, most general hospitals evaluate resident physicians, including all doctors, primarily based on their scientific research output. This evaluation emphasizes the publication of articles in high-impact journals and the attainment of national or provincial natural science funding. The volume of scientific research output significantly influences their ability to remain at the hospital and their prospects for promotion to attending physician. Consequently, this orientation compels residents to allocate a substantial portion of their time to scientific research, diverting their attention from clinical rotations. I once observed one of my residents in a state of exhaustion after completing a 24-hour night shift. Following this demanding shift, he was required to attend a scientific research group meeting organized by his supervisor, as well as participate in the hospital's resident training course. After fulfilling these obligations, he planned to return to the ward to continue documenting patient cases. Additionally, he had to contemplate the upcoming high rent due next month in Beijing, a city characterized by its high cost of living.

Therefore, it is essential to learn from the successful practices of the University of Chicago Pritzker School of Medicine and to integrate growth mindset-related training into the resident training system. This integration aims to enhance residents' abilities to navigate complex situations and solve intricate problems. More importantly, it fosters mental resilience in residents, equipping them to better manage stress and setbacks. The cultivation of a growth mindset is not only vital for residents but also for senior physicians, as we all encounter the complexities of the medical environment, uncertainties related to diseases, and the challenges of our own professional development. Effectively addressing these challenges necessitates the support of a growth mindset.

In my view, the learning and application of the successful practices of the University of Chicago Pritzker School of Medicine should extend beyond merely teaching courses on growth mindset to medical undergraduates and residents. It is essential to provide Personal and Professional Development opportunities for the resident group, as well as psychological counseling for medical undergraduates and residents who are experiencing emotional distress and significant psychological pressure. Additionally, residents should receive individualized career development plans tailored to their unique aptitudes. For those residents identified as having 'poor performance,' it is crucial to avoid hasty judgments; instead, we should assist them in analyzing the underlying reasons for their performance through face-to-face interviews and empathetic communication skills. Together, we should identify which challenges we can help them address and overcome. The medical education system must evolve. While imparting knowledge and skills to students is important, fostering physically and mentally healthy, optimistic residents is even more critical. Physicians in teaching hospitals naturally assume dual roles: that of a doctor and an educator. As we care for our patients, we must also prioritize the support and well-being of our medical students and residents. Attending to their well-being not only contributes to the development of future doctors but also positively impacts patient care.

The ancient Chinese philosopher Xunzi, a third-century BCE thinker, articulated in his work "Encouraging Learning", a masterpiece of Chinese argumentation, that "If you do not accumulate small steps, you cannot reach a thousand miles; if you do not gather small streams, you cannot form a river [19]." This encapsulates the idea that a journey of a thousand miles begins with a single step. In the context of future clinical teaching for medical undergraduates and residents, I believe that China's medical education system must undergo practical changes. It is essential to cultivate lifelong learning abilities, complex problem-solving skills, and mental resilience among medical students and young residents, alongside teaching clinical knowledge and skills. To foster a growth mindset in these individuals, we should draw lessons from the successful experiences of the University of Chicago Pritzker School of Medicine and engage in diversified international medical education exchanges, while ensuring that these approaches are tailored to the realities of Chinese medical education. We need to gain a comprehensive understanding of the perspectives of the majority of young medical students and residents born after 2000, actively listening to their voices and insights to develop practical solutions. Furthermore, enhancing the well-being of medical students and residents through growth mindset interventions also contributes to the well-being of patients. Importantly, all physicians, not just young medical students and residents, can benefit from adopting growth mindset practices to more effectively address the complex challenges they encounter in their careers.
